# Measures of fragmentation of rest activity patterns: mathematical properties and interpretability based on accelerometer real life data

**DOI:** 10.21203/rs.3.rs-3543711/v1

**Published:** 2023-11-06

**Authors:** Ian Meneghel Danilevicz, Vincent Theodoor van Hees, Frank van der Heide, Louis Jacob, Benjamin Landré, Mohamed Amine Benadjaoud, Séverine Sabia

**Affiliations:** 1 Université Paris Cité, INSERM, U1153, CRESS, Epidemiology of Ageing and Neurodegenerative Diseases, 10 Av de Verdun, 75010, Paris, France; 2 Accelting, Almere, the Netherlands; 3 Institut de Radioprotection et de Sûreté Nucléaire (IRSN), 31 Av Division Leclerc, 92260, Fontenay-Aux-Roses, France; 4 Department of Epidemiology and Public Health, University College London, London, United Kingdom

**Keywords:** Circadian rhythm, detrended fluctuation analysis, inter-daily stability, intradaily variability, transition probability, Whitehall II cohort

## Abstract

Accelerometers, devices that measure body movements, have become valuable tools for studying the fragmentation of rest-activity patterns, a core circadian rhythm dimension, using metrics such as inter-daily stability (IS), intradaily variability (IV), transition probability (TP), and self-similarity parameter (named α). However, their use remains mainly empirical. Therefore, we investigated the mathematical properties and interpretability of rest-activity fragmentation metrics by providing mathematical proofs for the ranges of IS and IV, proposing maximum likelihood and Bayesian estimators for TP, introducing the activity balance index metric, an adaptation of α, and describing distributions of these metrics in real-life setting. Analysis of accelerometer data from 2,859 individuals (age=60–83 years, 21.1% women) from the Whitehall II cohort (UK) shows modest correlations between the metrics, except for ABI and α. Sociodemographic (age, sex, education, employment status) and clinical (body mass index (BMI), and number of morbidities) factors were associated with these metrics, with differences observed according to metrics. For example, a difference of 5 units in BMI was associated with all metrics (differences ranging between −0.261 (95% CI −0.302, −0.220) to 0.228 (0.18, 0.268) for standardised TP rest to activity during the awake period and TP activity to rest during the awake period, respectively). These results reinforce the value of these rest-activity fragmentation metrics in epidemiological and clinical studies to examine their role for health. This paper expands on a set of methods that have previously demonstrated empirical value, improves the theoretical foundation for these methods, and evaluates their empirical worth in a large dataset.

## 1 Introduction

A large number of human behaviours and physiological functions follow a circadian rhythmicity, such as for examples sleep/wake cycles, body temperature, and hormonal levels [1]. Circadian regulation of these processes is critical to maintaining homeostasis; prolonged disruptions are detrimental to health [2, 3], highlighting the importance of precise, scalable measures of human circadian rhythm (CR). Accelerometers, devices that record acceleration of the part of the body to which they are attached, have emerged as valuable tools to measure dimensions of CR based on movements in free-living conditions [4, 5].

An important dimension of CR is the fragmentation of rest-activity patterns over several consecutive days [6, 7]. Over time several metrics have been proposed to quantity the rest-activity fragmentation using accelerometry data. The first and now commonly used metrics are inter-daily stability (IS) and intradaily variability (IV). IS provides information on how constant the rest-activity pattern is between days and IV quantifies the fragmentation of activity pattern between hours over the observation period [6, 8]. Later the transition probability (TP) has been proposed to measure the likelihood of transitioning from a state of rest to a state of activity, or vice versa [7, 9]. In parallel, the Detrended Fluctuation Analysis (DFA) [10] initially used in genomics has been used to identify hidden patterns where activity fluctuations are used as proxy for rest-activity fragmentation [11, 12]. In DFA, the self-similarity parameter, also known as the scaling exponent or α, is a key metric for description of time series, such as stationary and non-stationary time series, random noise and fractal noise, among others [13].

Although metrics of rest-activity fragmentation are increasingly used in the literature, mathematical properties of these metrics and their interpretation have not been entirely described. First, although the range of IS in [0, 1] and IV in [0, 2] has been suggested [14], no proper mathematical proof is available, limiting confidence in interpretability, particularly for extreme values. Second, [7, 9] have proposed different estimations of TP, both based on heuristic estimators, limiting their mathematical properties as compared to estimators based on maximum likelihood (ML) or Bayesian inference. In addition, the properties of these two estimators have not been compared. Third, interpretation of DFA-derived metrics is not straightforward. Finally, to our knowledge, only one study has shown the correlation between IS, IV, TP (based on [7] definition) and DFA within a unique sample, older adults living in residential facilities, limiting generalisability of findings [7].

In order to overcome limitations of the current evidence on rest-activity fragmentation metrics, the present study aims to 1) provide mathematical proof of the range of IS and IV, 2) propose a ML estimator, the gold standard of estimation, and a Bayesian estimator, with good properties, for TP, and 3) propose a new metric, that is a transformation of DFA-derived self-similarity parameter, named activity balance index (ABI), that reflects how balanced is the activity over several days, and 4) describe these metrics using data from the population-based Whitehall II accelerometer sub-study. All metrics discussed here may computed using our free codes available on GitHub^1^, and they will be included in the GGIR R’s package next versions.

## 2 Materials and methods

### 2.1 Preliminary definitions

Rest-activity fragmentation metrics are calculated based on different time series derived from raw acceleration signals (Table 1). These time series differ as a function of epoch lengths (eg minute or hour) and outcome considered (acceleration, dichotomous state (rest/activity), or proportion of the epoch in a state). Here are some preliminary definitions of these time series.

**Definition 1.** For each individual, a discrete stochastic process representing the intensity of movement over a time period [0, T] is defined as ${\left\{X_{t}\right\}}_{t\in T}$, with $X_{t}\in \left[0,\;\delta_{x}\right],\;\delta_{x}<\infty$, and t correspond to an epoch. The observed time series is vector represented as $x={\left(x_{1},\ldots ,x_{T}\right)}^{\prime }$. In the case of accelerometry data, $x_{t}$ corresponds to the acceleration recorded at the $t^{th}$ epoch and $\delta_{x}$ is the maximum measurable record for $x_{t}$.

**Definition 2.** For each individual, a second stochastic process representing the active (a) and rest (r) states is defined as ${\left\{Y_{t}\right\}}_{t\in T}$, with $Y_{t}\in \left\{r,\;a\right\}$, where $Y_{t}=a$ if $X_{t}>\delta_{y}$, and $\delta_{y}$ is the threshold which separates active and rest based on the amount of acceleration per epoch. The observed time series is a vector represented as $y={\left(y_{1},\ldots ,y_{T}\right)}^{\prime }$.

**Definition 3.** For each individual, a third stochastic process representing the proportion of active states per hour is defined as ${\left\{Z_{p}\right\}}_{p\in P}$, with $Z_{p}\in \left[0,\;1\right]$, where $Z_{p}=\delta_{z}^{-1}\sum_{t=1}^{\delta_{z}}\;I\left(y_{t+\delta_{z}\left(p-1\right)}=a\right)$, I(⋅) is a indication function equal to one if the condition is true and zero otherwise, $\delta_{z}$ is the number of epochs which build 1 hour (eg if one epoch correspond to 1 minute then $\delta_{z}=60$) p corresponds to an epoch of 1 hour, and P the total number of hours during the period [0, T]. The observed time series is a vector represented as $z={\left(z_{1},\ldots ,z_{P}\right)}^{\prime }$.

### 2.2 Data

**Figure 1:**
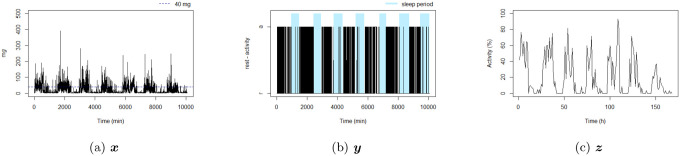
Example of three time series from the same individual

The Whitehall II study is an ongoing prospective cohort study established in 1985–1988 among 10308 British civil servants with clinical examinations every four-five years since inception. Written informed consent for participation was obtained at each contact. Research ethics approval was obtained from the University College London ethics committee (latest reference number 85/0938). An accelerometer measure was added to the 2012–2013 wave of data collection (age range 60 to 83 years) for participants seen at the London clinic and those living in the south-eastern regions of England who underwent clinical examination at home. Participants were requested to wear a tri-axial accelerometer (GENEActiv Original; Activinsights Ltd, Kimbolton, UK) on their non-dominant arm for nine consecutive 24-hour days. Accelerometer data, sampled at 85.7Hz and expressed relative to gravity $\left(1g=9.81m/s^{2}\right)$, were processed using GGIR v2.9–0 [15]. The Euclidean norm minus one (ENMO) of raw acceleration was calculated and corrected for calibration error and non-wear time. Average acceleration lower than 40 milligravity (mg) during the waking period was classified as rest corresponding to activities not classified as light or moderate-to-vigorous activities [16, 17]. Waking periods (ie, periods between waking and sleep onset) for each day were identified using an algorithm for sleep detection that has been previously described and evaluated [18]. Data from waking onset on day 2 to same time on day 8 were retained, resulting in 7 days of data. Non-wear time was detected using algorithm that has been previously described [19] and for the present study 2859 participants who wore the accelerometer over the full 7 consecutive days were included in analyses. For each individual, three time series were considered: the time series corresponding to the 1-minute epoch acceleration over 7 days $x_{t}$, t=1, …, 10080 (the number of minutes over 7 days) see definition 1; the time series corresponding to the 1-minute epoch active state over 7 days, $y_{t}$, t=1, …, 10080 see definition 2; and the time series corresponding to 1-hour proportion of active state over 7 days, $z_{p}$, p=1, …, 168 (the number of hours over 7 days) see definition 3. A summary of the three considered time series is available in Table 1, and an illustration is displayed in Figure 1.

For illustrative purposes, seven participant profiles were selected to highlight differences in metrics observed in real-life situations. They were chosen based on their lowest or highest value in the metrics.

Measures of socio-demographic (age, sex, education) and health-related (body mass index (BMI), prevalent morbidities) factors were also used. Education was categorised as zero if the individual has less than secondary school education and one otherwise. BMI was based on measured weight and height (kg/m^2^), and the number of prevalent morbidities was assessed using clinical examinations in the study and linkage to electronic health records and includes coronary heart disease, stroke, heart failure.

### 2.3 Inter-daily stability and intradaily variability

#### 2.3.1 Properties of IS and IV

IS measures how constant the rest-activity pattern is between days, this is a signal-to-noise measure, that is the ratio of the power of a signal (ordered by hour of the day) to the power of background noise (irrespective of the hours of the day) [8]. Considering that we measure H hours over D days, we have a total number of hours P=D×H over a full observation period. For IS, it is useful to organize the vector z from Definition 3 in a matrix form as

$$\dot{z}=\left[\begin{array}{ccc} z_{1,1} & \cdots & z_{1,H}\\ \vdots & & \vdots \\ z_{D,1} & \cdots & z_{D,H} \end{array}\right],$$

where $z_{d,h}$ is an element for the $d^{th}$ line and $h^{th}$ column, where d=1,…,D and h=1,…,H. IS is computed as

$$\text{IS}\left(z\right)=\frac{P\sum_{h=1}^{H}{\left({\bar{z}}_{h}-\bar{z}\right)}^{2}}{H\sum_{p=1}^{P}{\left(z_{p}-\bar{z}\right)}^{2}},$$

where ${\bar{z}}_{h}=\frac{1}{D}\sum_{d=1}^{D}z_{d,h}$ is the hour mean over the D days of measurement, and $\bar{z}=\frac{1}{P}\sum_{p=1}^{P}z_{p}$ is the general mean over the full observed period.

IV reflects the fragmentation of the rest-activity pattern over long periods of rest or activity, it measures the variability between consecutive hours (Figure 1c) [8]. IV is computed as

(1) $$\text{IV}\left(z\right)=\frac{P\sum_{p=2}^{P}{\left(z_{p}-z_{p-1}\right)}^{2}}{\left(P-1\right)\sum_{p=1}^{P}{\left(z_{p}-\bar{z}\right)}^{2}}.$$

Some mild conditions should be established to derive the properties of IS and IV metrics. They are:

(A1) $Z_{p}$ follows an autoregressive model of order 1 (AR(1) model) as
$$Z_{p}=\mu +\phi Z_{p-1}+\epsilon_{p},$$
  where μ is the mean of the stochastic process, ϕ is a fixed but unknown parameter with |ϕ|<1, $\epsilon_{p}$ is a Gaussian noise.
(A2) 0≤ϕ<1 and P→∞.

The assumption (A1) is required to define the IV range, because we need to determine the relationship between $Z_{p}$ and $Z_{p-1}$, and the AR(1) model is a very simple and flexible model, which can fit several different real situations. Although, we assume a stationary process, see unit root conditions in [20]. The assumption (A2) is imposed to guarantee a positive auto-correlation 0≤ϕ, and a long period of observation, P, of the time series.

**Theorem 1.** *Given a stochastic process ${\left\{Z_{p}\right\}}_{p\in P}$*, IS(z)∈[0, 1].

**Theorem 2.** *Given a stochastic process* ${\left\{Z_{p}\right\}}_{p\in P}$ *and under assumption (A1), IV(z)∈[0, ∞)*.

**Theorem 3.** *Given a stochastic process* ${\left\{Z_{p}\right\}}_{p\in P}$ *and under assumptions (A1) and (A2), ${\text{lim}}_{P\to \infty }\left(\text{IV}\left(z\right)\right)\in \left[0,\;2\right]$*.

The proof of these theorems is provided in the Supplementary material (Section 1).

#### 2.3.2 Interpretation of IS and IV

In the demonstration of Theorem 1 (Supplementary material - Section 1), we showed that 1) an IS value close to 1 reflects a rest-active pattern that is constant between days, as the signal is much stronger than the noise; 2) IS value close to zero corresponds to rest-activity pattern that is inconstant between days as the signal is weaker than the noise (Box 1). In the demonstration of Theorem 2 (Supplementary material - Section 1), we showed that 1) if ϕ goes to one (perfect autocorrelation), then IV goes to zero, reflecting a low rest-activity fragmentation between hours; 2) if ϕ goes to 0 (uncorrelated random noise), then IV goes to two, representing a high rest-activity fragmentation between hours; 3) in some specific cases, IV can be greater than 2, this can occur when the sample size P is too small, or ϕ<0 which may be seen in ultradian rhythm, which means that the rhythm cycle lasts less than a day [14], or in the case of use of high frequency data [21]. These statements agree with the previous claims given by [14] about IS and IV. Some authors as [22, 23, 24] use IV based on the x time series. In that case, the present properties do not hold anymore as the assumptions (A1) and (A2) are verified exclusively for z.

### 2.4 Transition probability

#### 2.4.1 Properties of TP

The TP in dichotomous stochastic processes represents the probability of a state change given a period of time spent in a specific state. In the case of changes in rest/activity state among human beings, a “U” shape association is expected between the TP and the time already spent in a specific state [7]. Indeed, if the time spent in one state is very short, the probability of transition to the alternate state should be relatively high as the current state is not consolidated; if the time spent in a given state is medium, then the chance of change is more likely to be small because the person is engaged in the specific behaviour. Finally if the time spent in a specific state is long, then the probability of transition is higher as people are unlikely stay in rest or activeness for a very long period of time.

**Definition 4.** For each individual, given a stochastic process determined by Definition 2 the TP from r to a given an uninterrupted period of rest with length equal to s is

$$\pi_{ra}\left(s\right)=𝒫\left(Y_{t}=a|Y_{t-1}=r,\ldots ,Y_{t-s}=r\right),$$

and the TP from a to r given an uninterrupted period of activity with length equal to s is

$$\pi_{ar}\left(s\right)=𝒫\left(Y_{t}=r|Y_{t-1}=a,\ldots ,Y_{t-s}=a\right).$$

**Definition 5.** For each individual, given a stochastic process determined by Definition 2 the TP from r to a given an uninterrupted period of rest with length equal to 1 is

(2) $$\pi_{ra}\left(1\right)=𝒫\left(Y_{t}=a|Y_{t-1}=r\right),$$

and the TP from a to r given one single epoch of activity is

(3) $$\pi_{ar}\left(1\right)=𝒫\left(Y_{t}=r|Y_{t-1}=a\right).$$

The two conditional probabilities from Definition 4 are proposed by [7]. The two TP from Definition 5 are special cases from the previous one and are highlighted by [9]. We aim to propose a ML estimator to TP because if the model assumptions are aligned, there is no better estimation than ML, so it is a gold standard. Although, if there is available knowledge, we can aggregate this information and build a Bayesian estimator that is even more accurate than ML. Beforehand, some notations need to be introduced for readability.

**Definition 6.** The $r={\left(r_{1},\ldots ,r_{n_{r}}\right)}^{\prime }$ is a $n_{r}$-vector that records the length of each consecutive bout of rest, where $n_{r}$ is the number of bouts of rest $\left(n_{r}\leq T\right)$ so that $r_{1}$ is the length of the first bout of rest, $r_{2}$ of the $2^{nd}$ bout of the rest, and $r_{n}$ the length of the last bout of rest. $T_{r}=\sum_{i=1}^{n_{r}}\;r_{i}$ is the total time of rest (in epochs unit), $r_{i}\in \left\{1,\ldots ,S_{r}\right\}$, $S_{r}$ is the duration of the longest bout of rest, and

$$N_{r}\left(s\right)=\overset{n_{r}}{\underset{i=1}{\sum }}I\left(r_{i}\geq s\right),$$

where $N_{r}\left(s\right)\geq N_{r}\left(s+k\right)$, for all k≥1. $N_{r}\left(s\right)$ is the number of bouts of rest of duration greater or equal to s. $\Delta_{r}\left(s\right)=N_{r}\left(s\right)-N_{r}\left(s+1\right)$ is the number of bouts of rest of duration equal to s. Some corrected measures are required to account for the fact that the last state is rest or not, as if the last state is rest, we cannot consider its transition to activity. They are

$$n_{r}^{*}=n_{r}-I\left(y_{T}=r\right),\;T_{r}^{*}=T_{r}-I\left(y_{T}=r\right),$$

$$N_{r}^{*}\left(s\right)=\{\begin{array}{ll} N_{r}\left(s\right), & \text{ if }y_{T}=a,\\ \sum_{i=1}^{n_{r}-1}I\left(r_{i}\geq s\right), & \text{ if }y_{T}=r, \end{array}$$

and $\Delta_{r}^{*}\left(s\right)=N_{r}^{*}\left(s\right)-N_{r}^{*}\left(s+1\right)$.

**Definition 7.** The $a={\left(a_{1},\ldots ,a_{n_{a}}\right)}^{\prime }$ is a $n_{a}$-vector that records the length of each consecutive bout of activity, where $n_{a}$ is the number of bouts of activity $\left(n_{a}\leq T\right)$. $T_{a}=\sum_{i=1}^{n_{a}}a_{i}=T-T_{r}$ is the total time of activity (in epochs unit), $a_{i}\in \left\{1,\ldots ,S_{a}\right\}$, $S_{a}$ is the duration of the longest bout of activity, and

$$N_{a}\left(s\right)=\overset{n_{a}}{\underset{i=1}{\sum }}I\left(a_{i}\geq s\right),$$

where $N_{a}\left(s\right)\geq N_{a}\left(s+k\right)$, for all k≥1. $N_{a}\left(s\right)$ is the number of bouts of activity of duration greater or equal to s. As for Definition 6, some corrected measures are required to account for the value of the last state. They are

$$n_{a}^{*}=n_{a}-I\left(y_{T}=a\right),\;T_{a}^{*}=T_{a}-I\left(y_{T}=a\right),$$

$$N_{a}^{*}\left(s\right)=\{\begin{array}{ll} N_{a}\left(s\right), & \text{ if }y_{T}=r,\\ \sum_{i=1}^{n_{a}-1}I\left(a_{i}\geq s\right), & \text{ if }y_{T}=a, \end{array}$$

and $\Delta_{a}^{*}\left(s\right)=N_{a}^{*}\left(s\right)-N_{a}^{*}\left(s+1\right)$.

Here is a list of assumptions:

(B1) The stochastic process ${\left\{Y_{t}\right\}}_{t\in T}$ is a Markov chain.
(B2) The stochastic process ${\left\{Y_{t}\right\}}_{t\in T}$ is stationary.
(B3) The stochastic process ${\left\{Y_{t}\right\}}_{t\in T}$ has a finite memory equal to s≥1.
(B4) Let us consider $n_{a}^{*}\gg N_{a}^{*}\left(S_{a}\right)$ and $n_{r}^{*}\gg N_{r}^{*}\left(S_{r}\right)$.

**Theorem 4.** *Given a stochastic process ${\left\{Y_{t}\right\}}_{t\in T}$*, *under assumptions (B1) and (B2), the ML estimator of* $\pi_{ra}\left(1\right)$ *and* $\pi_{ar}\left(1\right)$ *are* ${\hat{\pi }}_{ra}{\left(1\right)}_{ML}=\frac{n_{r}^{*}}{T_{r}^{*}}$ and ${\hat{\pi }}_{ar}{\left(1\right)}_{ML}=\frac{n_{a}^{*}}{T_{a}^{*}}$.

**Corollary 5.** *Given a stochastic process ${\left\{Y_{t}\right\}}_{t\in T}$*, *under assumptions (B1) and (B2), the Bayesian estimator of* $\pi_{ra}\left(1\right)$ *and* $\pi_{ar}\left(1\right)$ *are* ${\hat{\pi }}_{ra}{\left(1\right)}_{B}=\frac{n_{r}^{*}+\lambda }{T_{r}^{*}+\lambda }$ and ${\hat{\pi }}_{ar}{\left(1\right)}_{B}=\frac{n_{a}^{*}+\lambda }{T_{a}^{*}+\lambda }$, *for any λ>0*.

The proof of Theorem 4 is provided in (Supplementary material - Section 1) using as a main argument the properties of a Bernoulli stochastic process [25]. The proof of the Corollary 5 is a directly application from Theorem 4 for a Binomial model. Then the Beta-Binomial posterior estimator is a well known result [26, page 104].

The Bayesian estimator introduced in Corollary 5 always exists, even if any of $T_{r}$, $T_{r}$, $T_{a}$ or $T_{a}$ is zero. Whether λ=1 corresponds to the Uniform distribution prior, which gives an interesting interpretation, we aim that in a sequence of nights there is at least one epoch of activity and in a sequence of days there is a least one epoch of rest. Other values might be explored as λ=0.5 is the Horseshoe prior [27], or $\lambda =10^{-6}$ which returns a numerically insignificant difference between ML and Bayesian estimators. Larger values than one for λ may be not relevant in this context.

**Remark 1.** *Even without any assumption about the stochastic process in terms of memory and stability, some nonparametric measures are avaliable as the reciprocal average duration (RAD) of rest,* ${\text{RAD}}_{r}$, *and the RAD of activity,* ${\text{RAD}}_{a}$, *which are defined as*

(4) $${\text{RAD}}_{r}=\frac{n_{r}}{T_{r}},{\text{RAD}}_{a}=\frac{n_{a}}{T_{a}}.$$

*This metric appears in the work of* [28], *but it was used to approximate the target probabilities in* (2) *and* (3) *by* [9]. *If $y_{T}=r$*, *then* ${\text{RAD}}_{a}={\hat{\pi }}_{ar}{\left(1\right)}_{ML}$ *and* ${\text{RAD}}_{r}>{\hat{\pi }}_{ra}{\left(1\right)}_{ML}$ *as $T_{r}>n_{r}$*, *if $y_{T}=a$*, *then* ${\text{RAD}}_{r}={\hat{\pi }}_{ra}{\left(1\right)}_{ML}$ *and* ${\text{RAD}}_{a}>{\hat{\pi }}_{ar}{\left(1\right)}_{ML}$ *as $T_{a}>n_{a}$*.

Let us give a hypothetical example for a small sample size T=15 as $y={\left(a,\;a,\;a,\;r,\;r,\;a,\;r,\;a,\;a,\;a,\;r,\;r,\;a,\;r,\;r\right)}^{\prime }$ to illustrate the difference between ${\hat{\pi }}_{ra}{\left(1\right)}_{ML}$, ${\hat{\pi }}_{ra}{\left(1\right)}_{B}$ and ${\text{RAD}}_{r}$, as well as ${\hat{\pi }}_{ar}{\left(1\right)}_{ML}$, ${\hat{\pi }}_{ar}{\left(1\right)}_{B}$ and ${\text{RAD}}_{a}$, with hyperparameter λ=0.5. Thus $r={\left(2,\;1,\;2,\;2\right)}^{\prime }$ and $a={\left(3,\;1,\;3,\;1\right)}^{\prime }$, this corresponds to $n_{r}=4$, $n_{r}^{*}=3$, $n_{a}=4$, $n_{a}^{*}=4$, $T_{r}=7$, $T_{r}^{*}=6$, $T_{a}=8$, $T_{a}^{*}=8$. So we have 3 changes $\left(n_{r}^{*}\right)$ in 6 opportunities $\left(T_{r}^{*}\right)$ ie 50% of transitions from r to a by the ML estimator, the ${\text{RAD}}_{r}$ inflates this result to 57% by adding a transition for the last observation, but actually we don’t know what would happen in $y_{16}$. From a to r, ${\text{RAD}}_{a}$ and ML estimators are the same, and Bayesian estimator is also really close. For convenience, these values are available in Table 2.

**Theorem 6.** *Given a stochastic process ${\left\{Y_{t}\right\}}_{t\in T}$*, *under assumptions (B2) and (B3), the ML estimator of* $\pi_{ra}\left(s\right)$ *and* $\pi_{ar}\left(s\right)$ *are* ${\hat{\pi }}_{ra}{\left(s\right)}_{ML}=\frac{\Delta_{r}^{*}\left(s\right)}{N_{r}^{*}\left(s\right)}$ and ${\hat{\pi }}_{ar}{\left(s\right)}_{ML}=\frac{\Delta_{a}^{*}\left(s\right)}{N_{a}^{*}\left(s\right)}$, *for $s=1,\ldots ,S_{r}-1$*, *and $s=1,\ldots ,S_{a}-1$*, *respectively*.

The proof of Theorem 6 is provided in the Supplementary material (Section 1).

**Remark 2.** *The heuristic estimators proposed by* [7] *are*

$${\hat{\pi }}_{ar}{\left(s\right)}_{H}=\frac{N_{a}\left(s\right)-N_{a}\left(s+d\right)}{dN_{a}\left(s\right)},$$

*where $s=1,\ldots ,S_{a}-1$*, d≥1 *is the smallest value that do* $N_{a}\left(s\right)-N_{a}\left(s+d\right)>0$ *for ${\hat{\pi }}_{ar}{\left(s\right)}_{H}$*, *and*

$${\hat{\pi }}_{ra}{\left(s\right)}_{H}=\frac{N_{r}\left(s\right)-N_{r}\left(s+d\right)}{dN_{r}\left(s\right)},$$

*where $s=1,\ldots ,S_{r}-1$*, d≥1 *is the smallest value that do* $N_{r}\left(s\right)-N_{r}\left(s+d\right)>0$ *for ${\hat{\pi }}_{ra}{\left(s\right)}_{H}$*.

Note that if d=1 and $y_{T}=r$, then ${\hat{\pi }}_{ar}{\left(s\right)}_{H}={\hat{\pi }}_{ar}{\left(s\right)}_{ML}$ but otherwise they are different, and if d=1 and $y_{T}=a$, then ${\hat{\pi }}_{ra}{\left(s\right)}_{H}={\hat{\pi }}_{ra}{\left(s\right)}_{ML}$. Of note ${\hat{\pi }}_{ar}{\left(s\right)}_{ML}$, ${\hat{\pi }}_{ra}{\left(s\right)}_{ML}\in \left[0,\;1\right)$, though, ${\hat{\pi }}_{ar}{\left(s\right)}_{H}$, ${\hat{\pi }}_{ra}{\left(s\right)}_{H}\in \left(0,\;1\right)$, but at the price of biased estimates when d>1, which are more frequent as s increases [7]. Thus the positive tails of ${\hat{\pi }}_{ar}{\left(s\right)}_{H}$ and ${\hat{\pi }}_{ra}{\left(s\right)}_{H}$ are systematically affect by this bias.

#### 2.4.2 Summary statistics for TP

The conditional probabilities $\pi_{ar}\left(1\right)$ and $\pi_{ra}\left(1\right)$ are more convenient to interpret than $\pi_{ar}\left(s\right)$ and $\pi_{ra}\left(s\right)$. However, the assumption (B1) is much stronger than (B3), but the first it is not expected in accelerometer applications [7]. In the aim of summarizing the set of ${\hat{\pi }}_{ar}{\left(s\right)}_{H}$ and ${\hat{\pi }}_{ra}{\left(s\right)}_{H}$ in single values, [7] proposed a bounded average calculated by LOWESS smoothing over a range of s values where the TP is lower (determined a posteriori as the s values in the flat region of the “U” shape, see illustration in [7]. This method requires to determine the boundary of the s values for which there is not straightforward method.

Here we propose to summarize ${\hat{\pi }}_{ar}{\left(s\right)}_{ML}$ by a weighted mean as

(5) $${\bar{\pi }}_{ar}=\overset{S_{a}-1}{\underset{s=1}{\sum }}{\hat{\pi }}_{ar}{\left(s\right)}_{ML}\omega_{s},$$

where $\omega_{s}$ is a weight, $\sum_{s=1}^{S_{a}-1}\omega_{s}=1,{\bar{\pi }}_{ar}$ is a weight mean of ${\hat{\pi }}_{ar}{\left(s\right)}_{ML}$. The ${\hat{\pi }}_{ar}\left(s\right)$ is poorly estimated to large s because these events (large s) are rare [7]. Thus we propose to weight these values by a proportion of the sample size so that $\omega_{s}=\frac{N_{a}^{*}\left(s\right)}{k_{a}}$, $k_{a}=\sum_{s=1}^{S_{a}-1}N_{a}^{*}\left(s\right)$, then (5) is equal to

(6) $${\bar{\pi }}_{ar}=\overset{S_{a}-1}{\underset{s=1}{\sum }}\frac{\Delta_{a}^{*}\left(s\right)}{N_{a}^{*}\left(s\right)}\left(\frac{N_{a}^{*}\left(s\right)}{k_{a}}\right)=\frac{1}{k_{a}}\overset{S_{a}-1}{\underset{s=1}{\sum }}\frac{\Delta_{a}^{*}\left(s\right)}{1}=\frac{N_{a}^{*}\left(1\right)-N_{a}^{*}\left(S_{a}\right)}{\sum_{s=1}^{S_{a}}\left(N_{a}^{*}\left(s\right)\right)-N_{a}^{*}\left(S_{a}\right)}=\frac{n_{a}^{*}-N_{a}^{*}\left(S_{a}\right)}{T_{a}^{*}-N_{a}^{*}\left(S_{a}\right)}.$$

Analogously, ${\hat{\pi }}_{ra}{\left(s\right)}_{ML}$ is summarized by a weighted mean as

(7) $${\bar{\pi }}_{ra}=\cdots =\frac{n_{r}^{*}-N_{r}^{*}\left(S_{r}\right)}{T_{r}^{*}-N_{r}^{*}\left(S_{r}\right)}.$$

**Theorem 7.** *Given a stochastic process ${\left\{Y_{t}\right\}}_{t\in T}$*, *under assumptions (B2), (B3) and (B4), we observe that* ${\bar{\pi }}_{ar}\approx {\hat{\pi }}_{ar}{\left(1\right)}_{ML}$ *and ${\bar{\pi }}_{ra}\approx {\hat{\pi }}_{ra}{\left(1\right)}_{ML}$*. *Also, if* λ *is small, then* ${\bar{\pi }}_{ar}\approx {\hat{\pi }}_{ar}{\left(1\right)}_{B}$ *and ${\bar{\pi }}_{ra}\approx {\hat{\pi }}_{ra}{\left(1\right)}_{B}$*.

The proof of Theorem 7 is provided in the Supplementary material (Section 1). By the Theorem 7, we can say that estimated $\pi_{ar}\left(1\right)$ and $\pi_{ra}\left(1\right)$ are summary statistics of the functions of $\pi_{ar}\left(s\right)$ and $\pi_{ra}\left(s\right)$ for all values of s.

**Remark 3.** *We propose a Bayesian estimator that compared to ML or RAD, avoids to have values that can not be computed in case of no time spent in a state (denominator null). For an epidemiological motivation, we split these metrics by wake and sleep windows (that is the period between waking and sleep onset (wake), and between sleep and next waking for the day to start (sleep), respectively, as*

$${\text{TP}}_{ra,\text{w}}=\frac{n_{r,\text{w}}^{*}+\lambda }{T_{r,\text{w}}^{*}+\lambda },{\text{ TP}}_{ra,\text{ s}}=\frac{n_{r,\text{ s}}^{*}+\lambda }{T_{r,\text{ s}}^{*}+\lambda },$$

*the TP from rest to active period during the waking window, and the TP from rest to active period during the sleep window, respectively, and*

$${\text{TP}}_{ar,\text{w}}=\frac{n_{a,\text{w}}^{*}+\lambda }{T_{a,\text{w}}^{*}+\lambda },{\text{ TP}}_{ar,\text{ s}}=\frac{n_{a,\text{ s}}^{*}+\lambda }{T_{a,\text{ s}}^{*}+\lambda },$$

*the TP from active to rest period during the waking window, and the TP from active to rest period during the sleep window, respectively, where λ∈(0, 1]*, $n_{a,\text{w}}^{*}$ *is the number of bouts of activity during the awake time,* $n_{a,\text{s}}^{*}$is the number of bouts of activity during the sleep time, $n_{r,\text{w}}^{*}$is the number of bouts of rest during the awake time, $n_{r,\text{s}}^{*}$ is the number of bouts of rest during the sleep time, $T_{r,\text{w}}^{*}$is the total rest time during the awake time, $T_{r,\text{s}}^{*}$is the total rest time during the sleep time, $T_{a,\text{w}}^{*}$is the total activity time during the awake time, $T_{a,\text{s}}^{*}$is the total activity time during the sleep time. The star (*) *means minus one if it is not possible judge the last transition*.

#### 2.4.3 Interpretation of TP

When using a small λ and a long period of observation, higher ${\text{TP}}_{ra,\text{w}}$ corresponds to higher transition from rest to active period during the awake window, reflecting a more fragmented pattern of rest, higher ${\text{TP}}_{ar,\text{w}}$ corresponds to higher transition from active to rest period during the awake window, denoting a more fragmented pattern of activity. Similar interpretation applies to the metrics defined during the sleep window (Box 1). In case of absence of one state during a window period as for example no activity at all during the sleep window, the TP exists and transition from this missing state to the alternative state equals to 1 as it is likely that whether it happens the person moves to this state, the person will go back easily to the alternative state.

### 2.5 Detrended fluctuation analysis

#### 2.5.1 Introduction to DFA

The DFA is a powerful analytical tool for time series analysis initially proposed by [10] to analyse long-term correlation of nucleotides. More recently it has been used in the context of movement behaviour to quantity fractal fluctuations in activity over a range of time scales [12, 29]. In practise, it aims to evaluate to which extent the activity pattern (in terms of temporal and structural properties) is similar at different time scales. Estimating the self-similarity parameter allows differentiating stationary and nonstationary stochastic processes and identifying white, pink (fractal), or brown noise patterns. These key properties might be hidden in complex time series, but DFA is a way to reveal them.

**Figure 2:**
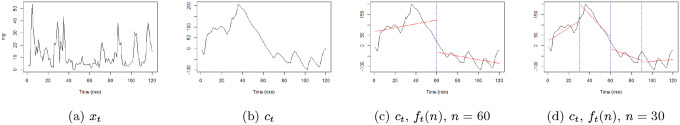
Example of DFA procedure

Let us consider a bounded stochastic process ${\left\{X_{t}\right\}}_{t\in T}$ from Definition 1. Take the accumulated signal with zero mean as

$$c_{t}=\overset{t}{\underset{i=1}{\sum }}\left(x_{i}-\bar{x}\right),t\leq T,$$

where $\bar{x}=T^{-1\;}\sum_{t=1}^{T}x_{t}$. Divide $c={\left(c_{1},\ldots ,c_{T}\right)}^{\prime }$ in B nonoverlapping boxes of equal n-size as $c_{1}={\left(c_{1},\ldots ,c_{n}\right)}^{\prime }$, $c_{2}={\left(c_{n+1},\ldots ,c_{2n}\right)}^{\prime }$, until $c_{B}={\left(c_{\left(B-1\right)n+1},\ldots ,c_{Bn}\right)}^{\prime }$. For each box, we fit a polynomial of order l, eg, the polynomial for the $j^{th}$ box is fit using ordinary least squares regression $f_{t}\left(n\right)={\hat{\beta }}_{0}+{\hat{\beta }}_{1}t+\cdots +{\hat{\beta }}_{l}t^{l}$, t=(j−1)n+1,…,jn. Note that $\beta ={\left(\beta_{0},\ldots ,\beta_{l}\right)}^{\prime }$ is different to each $j^{th}$ box and each n-size, consequently $f_{t}\left(n\right)$ depends of t and n. To detrend the integrated time series, ie, remove the trend of $c_{t}$, we take the difference of each pair $c_{t}$ and $f_{t}\left(n\right)$. For a given n-size box, the root mean square fluctuation is

(8) $$F\left(n\right)=\sqrt{\frac{1}{T}\overset{T}{\underset{t=1}{\sum }}{\left(c_{t}-f_{t}\left(n\right)\right)}^{2}},$$

which can be computationally obtained by [30]. Repeat the operation for a broad range of n-size box, eg, [31] recommend taking a sample on the grid between 4≤n≤T/4. The figure 2 display the steps of DFA for two n-size boxes, the first with 60 minutes (figure 2c) and the second with 30 minutes (figure 2d).

#### 2.5.2 Summary statistic for DFA and interpretation

Instead of displaying a function of F(n) for a grid of n, we can summarize this information by the self-similarity parameter. The root mean square fluctuation in (8) is proportional to the n-size, $F\left(n\right)\propto n^{\alpha }$, where α is called the scaling exponent or self-similarity parameter, which is estimated

$$\text{log}\left(F\left(n\right)\right)=\mu +\alpha \text{ log}\left(n\right)+\epsilon_{n},4\leq n\leq T/4,$$

where $\epsilon_{n}$ follows an independent Gaussian error, μ is an intercept, an ordinary least squares regression (OLS) was done to achieve $\hat{\alpha }$.

The interpretation of α is quite precise, but requires much mathematical jargon. Given a stochastic process as determined by Definition 1, the self-similarity parameter belongs to the range 0<α<1 for stationary stochastic processes, and 1<α<2 for nonstationary as proofed by [32]. Some critical values of the scaling exponent are of distinct mathematical importance as α=0.5 means that the stochastic processes is white noise, α=1 is related to pink or fractal noise, α=1.5 is the case of a random walk [33].

#### 2.5.3 Activity balance index: a new DFA-derived metric

Given previous empirical results, [34] hypothesized that many biological systems present a fractal nature, ie, α=1. A further hypothesis that healthy people presents fractal noise for heart and walking rates has been elaborated by [35]. In the context of activity behaviour, we have introduced a novel metric named ABI, that measures how balanced is the activity over the observed period, higher values reflect a more balanced pattern of activity. It is a transformation of $\hat{\alpha }$ as

(9) $$\text{ABI}\left(\hat{\alpha }\right)=\text{exp}\left\{\frac{-\left|\hat{\alpha }-1\right|}{\text{exp}\left(-2\right)}\right\},$$

where $\hat{\alpha }\in \left(0,\;2\right)$. If $\hat{\alpha }$ goes to one, then $\left|\hat{\alpha }-1\right|$ goes to zero and $\text{ABI}\left(\hat{\alpha }\right)$ goes to one. On the other direction, as $\hat{\alpha }$ goes to two or zero, which are the extremes for α [32], $\left|\hat{\alpha }-1\right|$ goes to one and $\text{ABI}\left(\hat{\alpha }\right)$ goes to 0.0006. The ABI has two advantages: it penalizes the scattering of $\hat{\alpha }$ in both directions and spreads its values over a large range between (0.0006, 1] or (0, 1] for simplicity.

We introduced the ABI that focusses on the fractal noise nature of the signal to evaluate how the activity is balanced over the observation period. If fractal noise represents an optimum balance for activity behaviour, then healthy individuals would present higher values for their ABI metric than unhealthy people (Box 1). Both $\hat{\alpha }$ and ABI are influenced by the choice of the epoch lengths, larger epoch values will naturally tend to smoothed acceleration signal, implying lower chance of observing a fractal noise (that is $\hat{\alpha }$ and ABI closer to one).

### 2.6 Strengths and limitations of IS, IV, TP and DFA

The strength and limitations of rest-activity fragmentation metrics are summarized in Box 1.

## 3 Results

A total of 4,880 individuals were invited to participate to the Whitehall accelerometer sub-study. Out of these, 4,282 agreed to wear the accelerometer and had no contraindications (allergy to plastic or travelling abroad). Among them, 2,859 individuals had complete data without any non-wear period for a continuous period of 7 days corresponding to a total of 10,080 epochs. The mean age of the participants was 69.2 years, with a standard deviation (SD) of 5.7 years. A total of 602 were women (21.1%), 1170 (40.9%) had less than secondary school education level, 495 (17.3%) were currently employed, and 1140 (39.9%) had at least one morbidity. The mean BMI in the study sample was 26.7 (SD=4.3) kg/m^2^.

**Figure 3:**
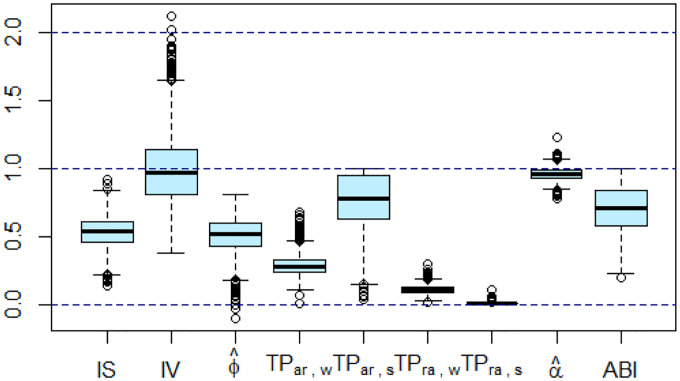
Boxplot of inter-daily stability (IS), intradaily variability (IV), estimated autocorrelation parameter of AR(1) model $\left(\hat{\phi }\right)$, transition probability (TP) from activity to rest during the awake period $\left({\text{TP}}_{ar,\text{w}}\right)$, TP from activity to rest during the sleep $\left({\text{TP}}_{ar,\text{s}}\right)$, TP from rest to activity during the awake $\left({\text{TP}}_{ra,\text{w}}\right)$, TP from rest to activity during the sleep $\left({\text{TP}}_{ra,\text{s}}\right)$, estimated self-similarity $\left(\hat{\alpha }\right)$, activity balance index (ABI)

Figure 3 shows the distribution of IS, IV, TP, $\hat{\alpha }$ and ABI in the total sample. All empirical ranges are within the theoretical ones proposed in Box 1. For IV, two individuals have a value that exceeds 2, these outliers correspond to two of the three individuals whose $\hat{\phi }$ value is not within the [0, 1] interval, suggesting a minority of cases with ultradian rhythm in the dataset.

When examining how rest-activity fragmentation metrics differ by sex (Table 3), we found that men have on average a less constant rest-activity pattern as denoted by smaller IS compared to women (0.529 vs 0.546, p=0.001). During the day, men tend to transition less from rest to active periods while during the night men are more likely to transition from active to rest period as indicated by lower ${\text{TP}}_{ra,\text{w}}\;(p<0.001)$ and higher ${\text{TP}}_{ar,\text{s}}\;(p<0.001)$ than in women. Finally, on average they tend to have a less balanced activity behavior than women as shown by lower $\hat{\alpha }\;\left(p=0.022\right)$ and ABI (p<0.001).

Less differences were observed as a function of age, although we found that older people tend to have a more fragmented rest-activity pattern (IV 1.039 vs 0.946 for age ≥ 70 vs < 70), tend to transition more from active to rest during waking periods (${\text{TP}}_{ar,\text{w}}$ 0.305 vs 0.273), and to transition less from rest to active periods during the day (${\text{TP}}_{ra,\text{w}}$ 0.098 vs 0.109), but more during the night (${\text{TP}}_{ra,\text{s}}$ 0.08 vs 0.007); all p<0.001.

Table 4 shows one fitted multivariate regression for each standardized rest-activity fragmentation metric. Being a woman, aged around 70 years old (see Figure 1 in Supplementary additional results for association with age), with lower educational level, not currently employed, having lower BMI and less prevalence morbidity were associated with a more constant rest-activity pattern (all p<0.05). The same variables (except for sex) were associated with IV, but in the opposite direction denoting less fragmented rest-activity pattern. ${\text{TP}}_{ar,\text{w}}$ is associated with all sociodemographic and health-related factors (except for sex and employment status), and ${\text{TP}}_{ar,\text{s}}$, in a complementary way, is only significantly associated with sex and employment status. Higher ${\text{TP}}_{ra,\text{w}}$ is associated with being a woman, lower BMI and less morbidities while higher ${\text{TP}}_{ra,\text{s}}$ is associated with higher BMI and more prevalent morbidities. Both $\hat{\alpha }$ and ABI are associated with all socio-demographic (except education) and health-related factors.

Table 5 presents Pearson’s correlation coefficients for IS, IV, TP, $\hat{\alpha }$, and ABI metrics. We observe one moderate correlation between $\hat{\alpha }$ and ABI that is expected as ABI is a transformation of $\hat{\alpha }$ and is not aimed to be used simultaneously. All the remaining correlations are considered fair or poor [36].

Figures 4 to 9 show the time series processes of individuals with extreme IS, IV, TP, and DFA values. In footnotes, a short description of what characterized these time series is provided. More figures are available in the Supplementary material (Section 2, Figures 2 to 5).

## 4 Discussion

This study provides theoretical ranges and guidance on the interpretation of rest-activity fragmentation metrics. We mathematically compared the heuristic estimators of TP proposed by [7] and [37] and proposed alternative ML and Bayesian estimators. We also proposed a transformation of DFA-derived self-similarity parameter, the ABI to reflect the balance of activity behaviours over the observation period. Finally, using accelerometer data from around 2,859 individuals aged 60 to 83 years, we showed that most of the correlations between IS, IV, TP and ABI were modest. We also found sociodemographic and health related differences in some of the rest-activity fragmentation metrics but not all, highlighting the fact that they measure different features.

We proposed Bayesian estimators of TP to estimate the chance to change from rest to active periods and vice versa, defined separately during the awake (day) and the sleep (night) windows. We observed as expected, a higher TP from active to rest period during the sleep window than during the awake period and, on the reverse, a higher TP from rest to active period during the awake window than during sleep period [38]. We applied these metrics to rest/activity state defined by threshold of acceleration [16, 17]. Empirical results are dependent on the method used to differentiate rest from activity states. These metrics might also be relevant using methods that differentiate sleep and wake states instead of rest and activity states. This might be particularly relevant to evaluate the fragmentation of sleep during the night.

**Figure 4:**
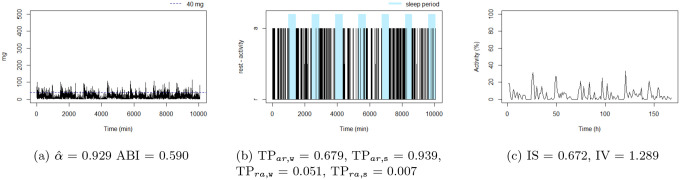
The sedentary: this individual presents the highest ${\text{TP}}_{ar,\text{w}}$. Note that the black blocks in the non-blue region of figure (b) are short, ie, this individual has short bouts of activity.

**Figure 5:**
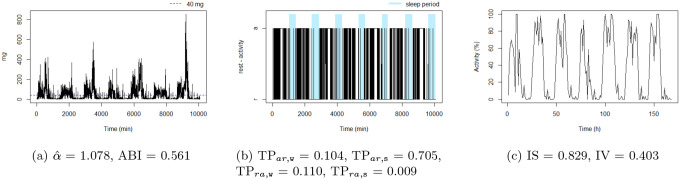
The active: this individual presents the lowest ${\text{TP}}_{ar,\text{w}}$. Note that the black blocks in the non-blue region of figure (b) are very long, ie, this individual has long bouts of activity.

**Figure 6:**
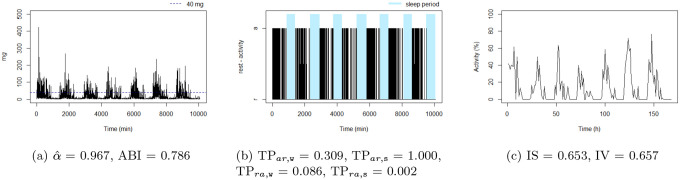
The good sleeper: this individual presents the lowest ${\text{TP}}_{ra,\text{s}}$ and a high ${\text{TP}}_{ar,\text{s}}$. Note that the black (white) blocks in the blue region of figure (b) are very brief (long), ie, during the night this individual almost does not display activity.

**Figure 7:**
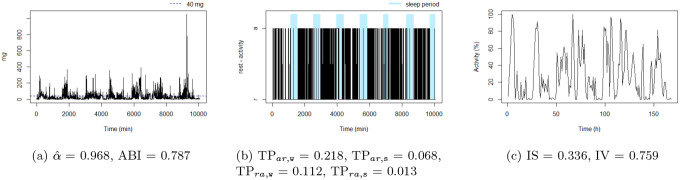
The insomniac: this individual presents the lowest ${\text{TP}}_{ar,\text{s}}$. Note some large black blocks in the blue region of figure (b), specially at the third and fifth sleep windows, ie, during the night this individual presents long periods of activity.

**Figure 8:**
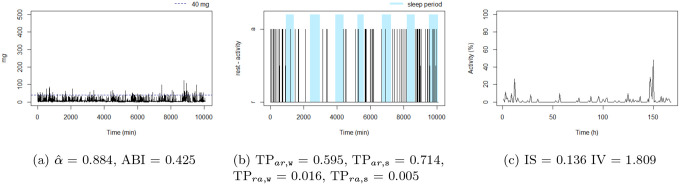
The unbalanced rest-activity person, this individual presents the lowest IS, a high IV, both $\hat{\alpha }$ and ABI low. Note the flat time series in figure (c) displays a comparatively weak rest-activity pattern and high rhythm fragmentation. Note that the time series in figure (a) seems very random (constant spikes without clear difference between day and night), denoting a stationary random noise, ie, low $\hat{\alpha }$ and ABI.

**Figure 9:**
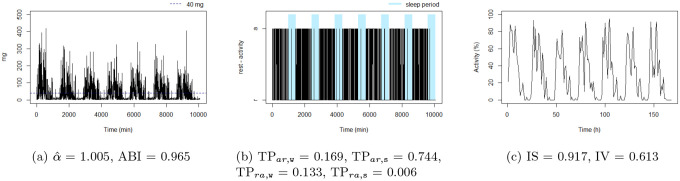
The balanced rest-activity person, this individual presents the highest IS, a low IV and a high ABI. Note the very regular waves in figure (c) denoting a high IS and low IV, ie, strong rest-activity pattern and low rhythm fragmentation. Note that the time series in figure (a) is very well balanced between smoothness and spikes, display a fractal noise and balanced motion, ie, $\hat{\alpha }$ and ABI close to one.

When comparing rest-activity fragmentation metrics using data from adults aged 60 to 83 years, we found low to moderate correlations among the variables (|r|<0.6), except for $\hat{\alpha }$ and ABI(r=0.789). Although calculated differently, these estimated correlations are in accordance with those found in the previous studies [7, 24, 39, 23, 40]. These modest correlations suggest that these metrics capture distinct features of individuals’ rest-activity patterns. The graphical analysis of the extreme cases of each metric (see Figures 4 to 9 and Figures 2 to 5 in Section 2 of the Supplementary material) displays several behavior profiles: sedentary, active, good sleeper, insomniac, (un)balanced rest-activity person, tireless person, ultradian rhythm person. There were differences in these metrics by sex, age, working status, BMI, and prevalent morbidities, implying the potential usefulness of these metrics for health outcomes.

The study has several strengths, including the use of both theoretical and empirical demonstrations of the range of the rest-activity fragmentation metrics, using a large sample size. The combination of the approaches increases the validity of our findings. Second, using multiple metrics in the same study population allows for a comprehensive comparison of these metrics. The study also has limitations. We used data from participants who had complete data for seven days. This may have resulted in a selection of the participants, highlighting the need to further investigate the impact of non-wear time on these metrics to allow the use of these metrics in a large sample. In addition, most participants were Caucasian and relatively healthy; whether results are valid in other ethnic subgroups requires further study. The empirical application is restricted to one type of device and should be replicated in other studies using different devices.

## 5 Conclusion

This study provided properties of rest-activity fragmentation metrics previously used and proposed new metrics. Their properties were evaluated using both theorical and empirical approach among more than 2800 older adults. Overall this study shows that the rest-activity fragmentation metrics examined in this paper - IS, IV, TP

## Figures and Tables

**Table 1:** Key information of each time series

Time series	$x={\left(x_{1},\ldots ,x_{T}\right)}^{\prime }$	$y={\left(y_{1},\ldots ,y_{T}\right)}^{\prime }$	$z={\left(z_{1},\ldots ,z_{P}\right)}^{\prime }$
Epoch	1 min	1 min	1 hour
Total time	T=10080	T=10080	P=168
Outcome	acceleration	rest/activity	proportion
Threshold	none	40 m*g*	40 m*g*

**Table 2:** Hypothetical example

Method	${\text{RAD}}_{r}$	${\hat{\pi }}_{ra}{\left(1\right)}_{ML}$	${\hat{\pi }}_{ra}{\left(1\right)}_{B}$	${\text{RAD}}_{a}$	${\hat{\pi }}_{ar}{\left(1\right)}_{ML}$	${\hat{\pi }}_{ar}{\left(1\right)}_{B}$
Numerator	4	3	3.5	4	4	4.5
Denominator	7	6	6.5	8	8	8.5
Estimation	0.57	0.50	0.54	0.50	0.50	0.53

**Table 3:** Mean (SD) and p-value of inter-daily stability (IS), intradaily variability (IV), transition probability (TP) from activity to rest during the awake period $({\text{TP}}_{ar,\text{w}})$, TP from activity to rest during the sleep $\left({\text{TP}}_{ar,\text{s}}\right)$, TP from rest to activity during the awake $\left({\text{TP}}_{ra,\text{w}}\right)$, TP from rest to activity during the sleep $\left({\text{TP}}_{ra,\text{s}}\right)$, estimated self-similarity parameter $\left(\hat{\alpha }\right)$, activity balance index (ABI)

	all	men	women		age < 70	age ≥ 70	
	mean (SD)	mean (SD)	mean (SD)	p-value	mean (SD)	mean (SD)	p-value
IS	0.533 (0.116)	0.529 (0.116)	0.546 (0.116)	0.001	0.534 (0.114)	0.531 (0.119)	0.442
IV	0.983 (0.251)	0.987 (0.254)	0.970 (0.241)	0.120	0.946 (0.237)	1.039 (0.261)	< .001
${\text{TP}}_{ar,\text{w}}$	0.285 (0.076)	0.285 (0.075)	0.286 (0.080)	0.931	0.273 (0.067)	0.305 (0.084)	< .001
${\text{TP}}_{ar,\text{s}}$	0.765 (0.194)	0.780 (0.193)	0.711 (0.189)	< .001	0.762 (0.194)	0.770 (0.195)	0.232
${\text{TP}}_{ra,\text{w}}$	0.105 (0.033)	0.104 (0.032)	0.110 (0.034)	< .001	0.109 (0.031)	0.098 (0.033)	< .001
${\text{TP}}_{ra,\text{s}}$	0.007 (0.005)	0.007 (0.005)	0.007 (0.004)	0.896	0.007 (0.004)	0.008 (0.006)	< .001
$\hat{\alpha }$	0.956 (0.046)	0.955 (0.048)	0.959 (0.037)	0.022	0.957 (0.047)	0.954 (0.043)	0.220
ABI	0.700 (0.169)	0.693 (0.172)	0.730 (0.152)	< .001	0.699 (0.171)	0.703 (0.166)	0.573

Note: all p-values come from ANOVA test.

**Table 4:** Association of socio-demographic and health-related factors with standardized rest-activity fragmentation metrics, results from multivariate linear regressions.

	IS		IV		$\hat{\alpha }$		ABI	
	Coeff.	(95% CI)	Coeff.	(95% CI)	Coeff.	(95% CI)	Coeff.	(95% CI)
Age *per 10 years*	2.535	(0.896, 4.175)*	−4.861	(−6.464, −3.258)*	3.721	(2.073, 5.369)*	2.890	(1.235, 4.546)*
Age^2^ *per 10 years*	−0.182	(−0.298, −0.066)*	0.373	(0.259, 0.487)*	−0.267	(−0.384, −0.150)*	−0.204	(−0.321, −0.086)*
Women	0.154	(0.064, 0.243)*	−0.063	(−0.150, 0.024)	0.110	(0.020, 0.200)*	0.219	(0.129, 0.309)*
Currently employed	−0.327	(−0.424, −0.229)*	0.136	(0.041, 0.232)*	−0.178	(−0.276, −0.080)*	−0.101	(−0.200, −0.003)*
High education	−0.112	(−0.188, −0.037)*	0.184	(0.110, 0.257)*	−0.064	(−0.140, 0.011)	−0.076	(−0.151, 0.000)
BMI *per 5 km/m*^2^	−0.168	(−0.211, −0.126)*	0.180	(0.139, 0.221)*	−0.152	(−0.194, −0.110)*	−0.127	(−0.169, −0.084)*
Morbidities	−0.085	(−0.135, −0.036)*	0.066	(0.018, 0.115)*	−0.073	(−0.123, −0.024)*	−0.056	(−0.106, −0.006)*
	${\text{TP}}_{ar,\text{w}}$		${\text{TP}}_{ar,\text{s}}$		${\text{TP}}_{ra,\text{w}}$		${\text{TP}}_{ra,\text{s}}$	
	Coeff.	(95% CI)	Coeff.	(95% CI)	Coeff.	(95% CI)	Coeff.	(95% CI)
Age *per 10 years*	−2.874	(−4.452, −1.295)*	−0.777	(−2.439, 0.884)	1.026	(−0.573, 2.624)	−0.828	(−2.479, 0.824)
Age^2^ *per 10 years*	0.234	(0.122, 0.346)*	0.059	(−0.059, 0.177)	−0.094	(−0.208, 0.019)	0.075	(−0.042, 0.192)
Women	−0.024	(−0.110, 0.062)	−0.346	(−0.437, −0.256)*	0.241	(0.154, 0.328)*	−0.016	(−0.106, 0.074)
Currently employed	−0.011	(−0.105, 0.083)	−0.106	(−0.204, −0.007)*	0.036	(−0.059, 0.131)	0.064	(−0.034, 0.162)
High education	0.088	(0.015, 0.160)*	0.023	(−0.053, 0.099)	−0.066	(−0.140, 0.007)	−0.053	(−0.128, 0.023)
BMI *per 5 km/m*^2^	0.228	(0.187, 0.268)*	−0.041	(−0.083, 0.002)	−0.261	(−0.302, −0.220)*	0.050	(0.007, 0.092)*
Morbidities	0.113	(0.066, 0.161)*	−0.030	(−0.080, 0.021)	−0.086	(−0.134, −0.038)*	0.114	(0.064, 0.164)*

Note:

* means significant at 0.95 confidence level, estimated coefficient (Coeff.), 95% confidence interval (95% CI), high education (secondary school or above), morbidities (number of prevalent morbidities among: coronary heart disease, stroke, heart failure, cancer, arthritis, chronic obstructive pulmonary disease, depression, Parkinson’s disease and dementia).

**Table 5:** Pearson’s correlation of inter-daily stability (IS), intradaily variability (IV), transition probability (TP) from activity to rest during the awake period $\left({\text{TP}}_{ar,\text{w}}\right)$, TP from activity to rest during the sleep $\left({\text{TP}}_{ar,\text{s}}\right)$, TP from rest to activity during the awake $\left({\text{TP}}_{ra,\text{w}}\right)$, TP from rest to activity during the sleep $\left({\text{TP}}_{ra,\text{s}}\right)$, estimated self-similarity parameter $\left(\hat{\alpha }\right)$ and activity balance index (ABI)

	IS	IV	${\text{TP}}_{ar,\text{w}}$	${\text{TP}}_{ar,\text{s}}$	${\text{TP}}_{ra,\text{w}}$	${\text{TP}}_{ra,\text{s}}$	$\hat{\alpha }$	ABI
IS	1.000	−0.483	−0.419	0.027	0.507	−0.053	0.333	0.323
IV		1.000	0.500	0.012	−0.525	0.111	−0.585	−0.513
${\text{TP}}_{ar,\text{w}}$			1.000	0.146	−0.475	−0.051	−0.447	−0.367
${\text{TP}}_{ar,\text{s}}$				1.000	−0.153	−0.371	0.073	0.044
${\text{TP}}_{ra,\text{w}}$					1.000	0.173	0.300	0.330
${\text{TP}}_{ra,\text{s}}$						1.000	−0.159	−0.130
$\hat{\alpha }$							1.000	0.789
ABI								1.000

## Data Availability

Data, protocols, and other metadata of the Whitehall II study are available to the scientific community either via the Whitehall II study data sharing portal (https://www.ucl.ac.uk/epidemiology-health-care/research/epidemiology-and-public-health/research/whitehall-ii/data-sharing).

## Box 1: Range, interpretability, strengths and limitations of rest-activity fragmentation metrics

Inter-daily stability (IS)

- Measure: how constant is rest-activity pattern between days.
- Range: [0, 1]
- Interpretation: higher values represent more constant rest-activity pattern.
- Strengths: it uses smooth data (over hours of the day) and is less sensitive to extreme values.
- Limitations: it depends on differentiation between rest/activity states for which no standards exist.

Intradaily variability (IV)

- Measure: the variability in activity hour by hour throughout the days.
- Range: [0, ∞)
- Interpretation: higher values represent more fragmented rhythm, values higher than two means ultradian rhythm or small sample size.
- Strengths: it uses smooth data (over hours of the day) making it less sensitive to extreme values.
- Limitations: it depends on differentiation between rest/activity states for which no standards exist.

Transition probability (TP)

- Measure: the TP from rest to activity (or activity to rest).
- Range: (0, 1]
- Interpretation:
  - ${\text{TP}}_{ra,\text{w}}$: higher values correspond to higher TP from rest to activity when in a rest period during the day, denoting a more fragmented rest/sedentary behaviour during the day.
  - ${\text{TP}}_{ra,\text{s}}$: higher values correspond to higher TP from rest to activity when in an rest period during the night, denoting a more fragmented rest during the night.
  - ${\text{TP}}_{ar,\text{w}}$: higher values correspond to higher TP from activity to rest when in an active period during the day, denoting a more fragmented activity pattern during the day.
  - ${\text{TP}}_{ar,\text{s}}$: higher values correspond to higher TP from activity to rest when in an active period during the night, denoting a higher propensity to go back to rest when active during the night.
- Strengths: it is based on dichotomous state (rest or activity) at the bout level, making it less sensitive to extreme values. It is defined separately during awake (day) and sleep (night) periods, allowing inference on the relevance of fragmentation of rest and activity separately for both periods.
- Limitations: it depends on a cut-point to differentiate rest and activity states.

Self-similarity parameter (α)

- Measure: the self-similarity of acceleration signal over the observation period.
- Range: (0, 2)
- Interpretation: values in the range (0, 1) means stationary motion behavior. Values in the range (1, 2) means nonstationary motion behavior. There are critical points as: 0.5 means random noise, 1 is fractal noise and 1.5 is random walk.
- Strengths: it considers the full activity distribution and is not dependent on the choice of a cut-point to differentiate rest-activity.
- Limitations: it is sensitive to extreme values that could be observed in the data. The interpretation requires mathematical knowledge.

Activity balance index (ABI)

- Measure: how the activity is balanced over the observation period.
- Range: (0, 1]
- Interpretation: higher values represent more balanced motion behaviour.
- Strengths: it considers the full activity distribution and is not dependent on the choice of a threshold to differentiate rest and activity. It is easy to interpret.
- Limitations: it is sensitive to extreme values that could be observed in the data.

## Footnotes

1 For printed version: https://github.com/iandanilevicz/frag_metrics
